# Strategies for the Development of pH-Responsive Synthetic Polypeptides and Polymer-Peptide Hybrids: Recent Advancements

**DOI:** 10.3390/polym13040624

**Published:** 2021-02-19

**Authors:** Cintya Dharmayanti, Todd A. Gillam, Manuela Klingler-Hoffmann, Hugo Albrecht, Anton Blencowe

**Affiliations:** 1 Applied Chemistry and Translational Biomaterials Group, Clinical and Health Sciences, University of South Australia, Adelaide, SA 5000, Australia; dhaig001@mymail.unisa.edu.au (C.D.); Todd.Gillam@unisa.edu.au (T.A.G.); 2 Surface Interactions and Soft Matter Group, Future Industries Institute, University of South Australia, Mawson Lakes, SA 5095, Australia; 3 Future Industries Institute, University of South Australia, Adelaide, SA 5001, Australia; Manuela.Klingler-Hoffmann@unisa.edu.au; 4 Drug Discovery and Development Group, Clinical and Health Sciences, University of South Australia, Adelaide, SA 5000, Australia; Hugo.Albrecht@unisa.edu.au

**Keywords:** pH-responsive, synthetic polypeptide, polymer-peptide hybrid, stimuli-responsive, drug delivery

## Abstract

Synthetic polypeptides and polymer-peptide hybrid materials have been successfully implemented in an array of biomedical applications owing to their biocompatibility, biodegradability and ability to mimic natural proteins. In addition, these materials have the capacity to form complex supramolecular structures, facilitate specific biological interactions, and incorporate a diverse selection of functional groups that can be used as the basis for further synthetic modification. Like conventional synthetic polymers, polypeptide-based materials can be designed to respond to external stimuli (e.g., light and temperature) or changes in the environmental conditions (e.g., redox reactions and pH). In particular, pH-responsive polypeptide-based systems represent an interesting avenue for the preparation of novel drug delivery systems that can exploit physiological or pathological pH variations within the body, such as those that arise in the extracellular tumour microenvironment, intracellularly within endosomes/lysosomes, or during tissue inflammation. Here, we review the significant progress made in advancing pH-responsive polypeptides and polymer-peptide hybrid materials during the last five years, with a particular emphasis on the manipulation of ionisable functional groups, pH-labile linkages, pH-sensitive changes to secondary structure, and supramolecular interactions.

## 1. Introduction

**Figure 1:**
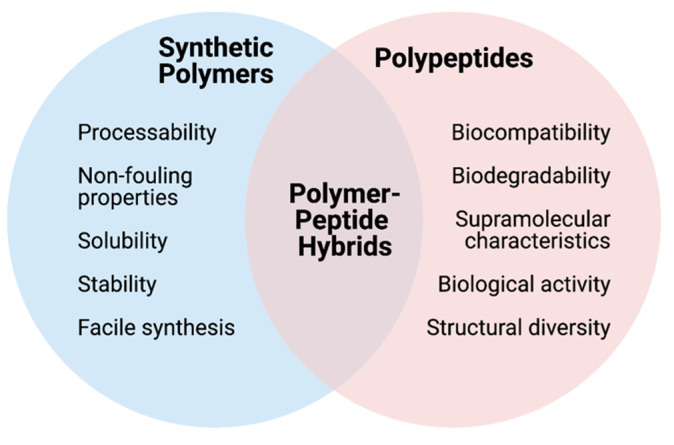
Respective contributions of synthetic polymers and polypeptides towards polymer-peptide hybrid materials.

Polypeptides are generally defined as short chains of amino acids linked to one another by covalent peptide bonds. Endogenously produced polypeptides are vital for a vast range of biological functions, from blood glucose regulation and osmoregulation to protection of cells from oxidative damage [1,2,3]. In recent decades, various synthetic polypeptides and polymer-peptide hybrids have been prepared with the aim of harnessing the biocompatibility, biodegradability and versatility of polypeptides for diverse applications in drug delivery and theranostics [4,5,6,7,8,9,10,11,12,13]. Herein, synthetic polypeptides refer to those that are produced through the ring-opening polymerization of amino acid *N*-carboxyanhydrides (NCAs) [14,15,16]. Contrastingly, polymer-peptide hybrids refer to materials containing both a synthetic polymer and synthetic peptide component, such as poly(ethylene glycol)-polypeptide block copolymers (PEG-*b*-polypeptides), which can be readily prepared through NCA polymerization using polymeric macroinitiators. Polymer-peptide hybrid materials elegantly combine the processability and versatility of conventional synthetic polymers with the biocompatibility and supramolecular characteristics of polypeptides [17]. In addition to their inherent biocompatibility, polypeptides and polymer-peptide hybrids also confer further advantages over their synthetic polyester and polycarbonate counterparts; namely their ability to form complex secondary structures, including coils, α-helices and β-sheets [18,19,20]. The selection and sequence of particular amino acid(s) can provide a means of controlling the overall hydrophilic-lipophilic balance of a polypeptide [21], allowing for the generation of unique supramolecular structures that can be difficult to attain using traditional synthetic monomers alone [14,20]. The peptide sequence, as well as the overall net charge of the polypeptide, can also help to facilitate specific biological interactions [9,22,23,24]. Finally, as a result of the structural diversity of naturally occurring amino acids, along with recent advances in the development of non-natural functional amino acid NCAs, polypeptides can feature a diverse array of functionalities, including (though not limited to) carboxyl, hydroxyl, amino, alkyne, alkene, saccharide and azido moieties, that are able to act as handles for further synthetic modifications [25,26,27,28,29]. The respective contributions of synthetic polymers and polypeptides towards polymer-peptide hybrid materials is summarized in Figure 1.

The use of synthetic polypeptides and polymer-peptide hybrids has emerged as an attractive approach towards the development of novel drug delivery systems with appealing physicochemical properties. For instance, polymer-peptide hybrid nanoparticles composed of PEG-*b*-poly(aspartic acid) and PEG-*b*-poly(glutamic acid) were utilised for the delivery of doxorubicin and a camptothecin derivative (SN-38), respectively, and demonstrated promising clinical characteristics in Phase I trials [30,31]. While these nanoparticles can facilitate accumulation of chemotherapeutics in tumour tissue passively via the enhanced permeation and retention (EPR) effect [32], the addition of stimuli-responsivity has the potential to further improve delivery to the target site [33]. A variety of stimuli have been investigated in the development of stimuli-responsive drug delivery systems, including temperature, light, redox reactions, and pH [9,34,35,36].

Among these, pH-responsivity is commonly employed due its potential applications in tumour-targeted drug delivery [37,38,39,40]. It is well established that the pH of the tumour extracellular microenvironment (6.4–6.8) and endosomal/lysosomal compartments (4–6.5) are significantly lower than normal physiological pH (7.4) [41,42]; a feature that can be exploited in the design of pH-responsive drug delivery systems [22,43,44]. Nanoparticles can be internalised into cells through a variety of endocytic mechanisms, where their uptake is greatly influenced by their size, shape, surface charge and surface chemistry [45,46,47]. Following cell internalisation, pH-responsive nanoparticles can be encapsulated within endosomal/lysosomal compartments and, on exposure to the acidic environment, release their cargo intracellularly. For example, ultra pH-sensitive tumour-imaging nanoprobes have been developed that were able to switch ‘on’ upon exposure to the acidic tumour milieu (pH 6.5–6.8) and endocytic organelles (pH 5.0–6.0), resulting in improved tumour detection and visualisation compared to conventional imaging probes [48].

Aside from tumour-targeting, pH-responsivity also has relevance to various other biomedical applications, ranging from hydrogels and wound dressings, to site-specific drug carriers [18,49,50], due to pH variations that exist at the organ (e.g., stomach), tissue (e.g., wound tissues), and subcellular (e.g., endosome) levels. These pH-responsive characteristics can be manipulated to trigger changes in the size, shape, surface charge and disassembly of drug delivery systems, offering a powerful strategy towards the controlled release of encapsulated cargo. Hence, the development of pH-responsive platforms has huge implications in improving the delivery of drugs to targeted sites, allowing for increased treatment efficacy.

**Figure 2:**
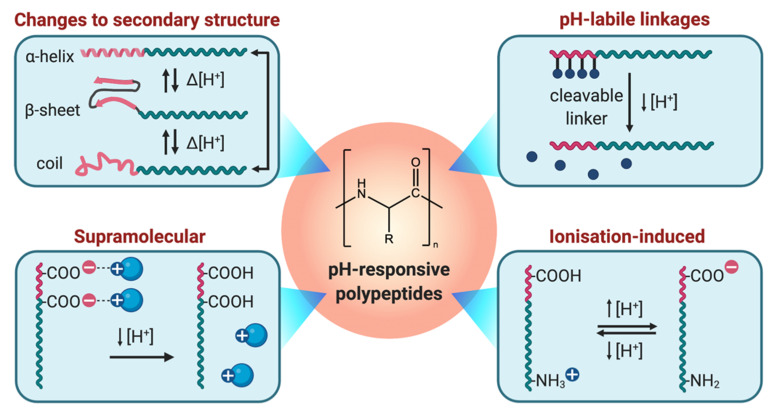
Strategies to prepare pH-responsive polypeptides and polymer-peptide hybrids.

This pH-responsivity, in combination with the aforementioned advantages of polypeptide-based materials, can give rise to targeted delivery systems that have the potential to reduce off-target side effects through the use of biodegradable components that are designed to respond to specific stimuli [16,51]. The ideal properties of a pH-responsive polypeptide-based drug delivery platform include structural stability until triggered by a stimulus, well-defined pH-responsivity, biocompatibility, and rational preparation via facile synthetic procedures. Thus, this review aims to highlight recent advancements in the field within the last five years for the preparation of pH-responsive synthetic polypeptides and polymer-peptide hybrids, with a particular focus on four key strategies that can be employed to achieve desirable pH-responsivity (Figure 2).

## 2. Overview of pH-Responsive Polypeptide and Polymer-Peptide Hybrid Preparation

### 2.1. Ionisation-Induced pH-Responsivity

**Figure 3:**
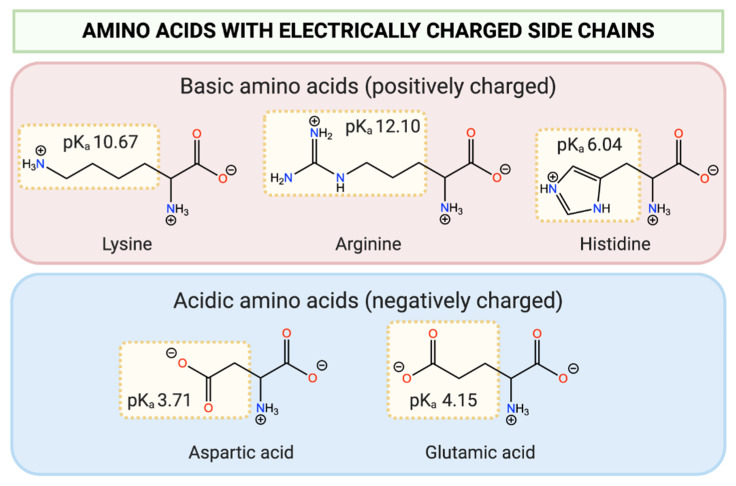
Structure of common basic and acidic amino acids, with the pK_a_ values of side chain functionalities shown (adapted from [52]).

In light of their structural diversity, amino acids can be categorised into distinct groups based on the nature of their side chain. With respect to pH-responsivity, amino acids with charged side chains are of particular interest. These side chain moieties can undergo ionisation based on their pK_a_ and in response to the pH of the surrounding environment. Lysine, histidine, and arginine are examples of amino acids that possess basic side chain functionalities that can become positively charged at pH values below their pK_a_. Contrastingly, aspartic acid and glutamic acid possess acidic carboxylic acid side chains that become negatively charged at pH values above their pK_a_ (Figure 3).

One of the simplest and most effective means to achieve pH-responsivity in polypeptide-based systems is through careful choice of the amino acid(s), as the ionisation state of the functional groups will dictate the overall hydrophilicity, and therefore physicochemical properties (i.e., aggregation, disassembly, swelling), of the system.

Histidine is frequently incorporated into pH-responsive polypeptide-based systems, as the pK_a_ of the imidazole group (~6.0) is biologically relevant for tumour targeting applications; the relative acidity of the tumour microenvironment causes protonation of the imidazole functionality. For example, Johnson et al. reported the preparation of pH-responsive vesicles composed of a poly(oligoethylene glycol acrylate)-*b*-poly(L-lysine)-*b*-poly(L-histidine) triblock copolymer for intracellular tumoural drug release [7]. In this ‘dual-hydrophilic’ system, the poly(L-histidine) block formed the inner hydrophobic bilayer and was responsible for the observed pH-responsivity. Reducing the pH from 7.4 to 5.5 resulted in swelling of the vesicle, attributed to protonation and charge repulsion of the imidazole functionalities under acidic conditions. In turn, this led to an improvement in doxorubicin release at pH 5.5 compared to pH 7.4 after 72 h (approximately 80% vs. 60%, respectively). At pH 5.5, over 50% of the drug was released within the first 24 h, followed by a gradual reduction in release rate. In comparison, drug release at pH 7.4 began to plateau after 50 h, and around 30% of the encapsulated doxorubicin still remained entrapped within the vesicles even after 72 h. Interestingly, increasing the length of the poly(L-histidine) block appeared to increase the diffusivity of the drug through the vesicle membrane at both pH values [7].

The ionisation of poly(L-histidine) at acidic pH values has been similarly exploited in a number of recently developed tumour-targeted systems [53,54,55], which exhibit improved cellular uptake, increased cytotoxicity, and, in some cases, also utilise the proton sponge effect of poly(L-histidine) to trigger effective endosomal/lysosomal escape. Generally, incorporation of a poly(L-histidine) block is an attractive means to achieve pH-responsivity due to the simplicity of the pH-response mechanism (functional group ionisation) and its facile synthesis; poly(L-histidine) blocks can easily be introduced into polymer materials through ring-opening polymerisation of the corresponding NCA with amine-terminated polymer macroinitiators. However, there are limitations to the pH-range that can be targeted using poly(L-histidine), which evidently lies around the pK_a_ of the imidazole group, and other amino acids with differing pK_a_ values must be used to target other pH ranges.

Acidic amino acids, such as aspartic and glutamic acid, are also commonly used to introduce pH-responsivity to polypeptide-based systems, particularly in the preparation of hydrogels. For instance, Bao et al. described the preparation of a pH-responsive hydrogel based on a four-armed PEG-*b*-poly(L-glutamic acid) copolymer, for potential applications in peptide and protein drug delivery [50]. The porous three-dimensional structure and high water content of hydrogels, in combination with their ability to offer protection against premature proteolytic degradation, makes them an appealing candidate for the delivery of protein therapeutics. Decreasing the pH of the solution to 3.0, significantly below the polymer’s apparent pK_a_ value of 6.5, resulted in aggregation and hydrogel formation due to protonation of the carboxylic acid groups and subsequent formation of physical cross-linking points between the poly(L-glutamic acid) chains. Contrastingly, gelation was not observed at pH values above 4.0 due to deprotonation of the carboxylic acid groups, increasing the solubility of the polymer and hindering gel formation. Insulin release from the hydrogels after 72 h was found to be minimal in an artificial gastric environment (pH 1.2; <20% release) but was significantly increased in an artificial intestinal environment (pH 6.8; 100%) [50].

Similarly, Sattari et al. prepared pH-responsive hybrid hydrogels through the use of poly(L-aspartic acid) cross-linked by graphene nanosheets and poly(acrylamide-*co*-acrylic acid), loaded with inorganic nanoparticles [56]. At pH 2.1, the swelling ratios of the hydrogels were negligible (<12%) but increased significantly at pH 7.4 (>120%). This was attributed to electrostatic repulsion between the anionic carboxylate groups of the poly(L-aspartic) acid component, resulting in an increase in hydrogel porosity and a subsequent increase in water uptake and swelling [56]. In agreement with these findings, the cumulative release of curcumin from the hydrogels was higher at pH 7.4 than at pH 2.1. Other than hydrogels, pH-responsive polypeptide-based nanoparticles have also been developed. For instance, Luan and co-workers prepared PEG-*b*-poly(*ε*-caprolactone)-*b*-poly(L-glutamic acid) nanoparticles loaded with doxorubicin and verapamil that exhibited ionisation-induced disassembly at pH 5.0 [57].

Polypeptide-based systems containing both acidic and basic amino acids in combination can also be used to confer tuneable pH-responsivity, through modification of the ratio between the positive and negatively charged components. Along this vein, Yang et al. reported the preparation of pH-responsive poly(L-lysine) and poly(L-glutamate)-grafted mesoporous silica nanoparticles (MSNs) [21]. The polypeptides were used as nanovalves to control drug release, where the overall net charge could be controlled to block or expose channel openings in the MSN. Due to the opposite charges of poly(L-lysine) and poly(L-glutamate) at physiological pH (7.4) (positive and negative, respectively) the polypeptides were able to self-assemble on the surface of the MSNs through electrostatic interactions, blocking the pores and preventing drug release. Indeed, only 7% of the encapsulated dye was released at neutral pH in the MSNs modified with a mixture of poly(L-lysine) and poly(L-glutamate). Reducing the pH below this value (4.5–6.5) resulted in an increase in the net positive charge, due to an increased proportion of ionised poly(L-lysine) and a decreased proportion of ionised poly(L-glutamate). In turn, this reduced the electrostatic interactions, disassembling the nanovalves and increasing the rate of drug release.

Aside from exploiting the pre-existing functional groups of the amino acids, further synthetic modifications can be introduced to attach acidic or basic functionalities to the polypeptide side chain to impart pH-responsivity. This strategy was explored by Shuai and co-workers, where the poly(L-aspartic acid) component of a diblock copolymer was functionalised with *N*,*N*-diisopropylamino ethylamine [58]. The ionisation state of this tertiary amine below pH 5.0 could be exploited to govern the disassembly of the vesicles. Indeed, release of doxorubicin was substantially faster at pH 5.0 compared to pH 7.4 after 24 h (>80% vs. 36%, respectively) due to protonation of the amine group [58]. Another tertiary amine, *N,N*-dimethylaminoethyl acrylate, was similarly attached to the side chain of poly(L-cysteine) via a thiol-ene reaction to achieve pH-responsive behaviour, resulting from protonation below pH 7.5 [59].

In an earlier study, Hammond and co-workers functionalised poly(γ-propargyl L-glutamate) with various azidoamine derivatives, including ethanamine (1°), *N*-methylethanamine (2°) and dimethylethanamine, diisopropylamine, and diethylamine (3°), through an alkyne-azide click reaction [60]. All of the investigated polymers were found to exhibit a similar buffering capacity between pH 5.0–7.35, regardless of the order of amine substitution (1°, 2° or 3°). While the polypeptides grafted with 1°, 2° and dimethyl-substituted 3° amines were water soluble between pH 3.0–10.5, the diisopropylamine and diethylamine-grafted polypeptides precipitated from solution at basic pH values due to deprotonation of the amine groups, and a subsequent reduction in polymer solubility. The polymers were complexed with siRNA at pH 5.5 and pH 7.4, and Ribogreen was used as a dye to investigate the complexation efficiency. The polypeptides functionalised with 1° amines demonstrated the highest complexation ability, requiring a two-fold lower charge ratio. Using a 4:1 charge ratio of polymer to siRNA, all amine-functionalised polymers were able to prevent dye access into the siRNA complex at pH 5.5. On increasing the pH to 7.4, deprotonation of the amines resulted in the formation of more permeable complexes, leading to higher dye penetration. This increased permeability was particularly evident in the polypeptides grafted with dimethylethanamine, diethylamine, and diisopropylamine [60].

### 2.2. pH-Responsivity Triggered by Functional Group Cleavage

Another strategy used to impart pH-responsivity into synthetic polypeptide-based systems is through attachment of cleavable functional groups through pH-labile linkages. These covalent linkages are designed to be stable at physiological pH but undergo cleavage in acidic pH ranges. The pH-labile linkages discussed in this review are summarised in Table 1.

**Figure 4:**
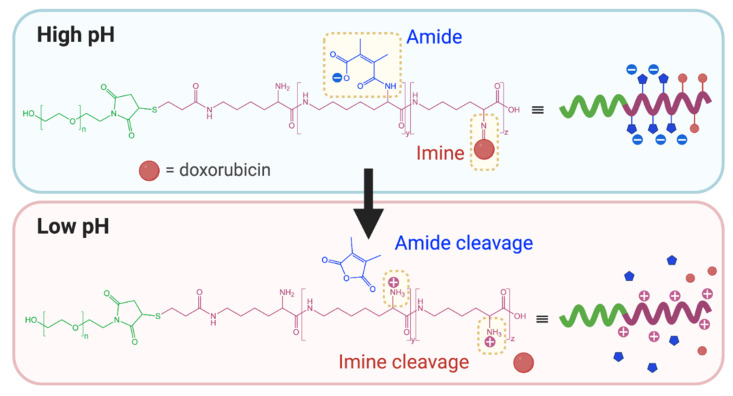
Dimethyl maleic anhydride and doxorubicin grafted to PEG-*b*-ɛ-poly(L-lysine) through amide and imine bonds, respectively, prepared by Guo et al. At pH 5.0–6.8, cleavage of the pH-labile amide and imine bonds results in charge reversal and drug release (adapted from [61]).

This approach was recently applied by Zhang et al. in the preparation of redox and pH-responsive nanoparticles, through attachment of dimethylmaleic anhydride (DMMA) to the free amine groups of poly(L-lysine) through a cleavable amide bond [9]. The lability of this amide bond is due to the presence of methyl substituents that generate steric hinderance in the ring-opened form. Under acidic conditions, the anhydride form is preferred and ring closing results in cleavage of the amide linker. Contrastingly, attachment of succinic anhydride (a non-substituted derivative) demonstrated no observable cleavage and exhibited a lack of pH-responsive behaviour. Incubation of the DMMA-grafted nanoparticles at pH 6.5 resulted in cleavage of the amide bond to liberate the free protonated amine groups of poly(L-lysine), coupled with a shift in zeta potential from negative to positive. Once stripped of DMMA, the nanoparticles also demonstrated higher cellular internalisation as a result of their increased surface potential. Guo et al. and Han et al. have also reported the preparation of pH-responsive PEG-*b*-ɛ-poly(L-lysine) and poly(L-lysine)-*b*-poly(L-leucine) nanoparticles, respectively, through attachment of DMMA [61,64]. In addition to the pH-labile amide bonds, Guo et al. also attached doxorubicin to PEG-*b*-ɛ-poly(L-lysine) through cleavable imine bonds (Figure 4) [61]. In both cases, cleavage of the DMMA group and subsequent charge reversal led to improved cellular uptake, highlighting functional group cleavage as an interesting approach to improve cell interactions. Notably, the PEG-*b*-ɛ-poly(L-lysine) nanoparticles dually loaded with doxorubicin and lapatinib demonstrated a markedly improved *in vivo* safety profile compared to administration of the free drugs [61].

Similarly, Tao et al. grafted dialkyl maleic anhydride moieties to PEG-*b*-poly(L-lysine) via amide linkages to generate pH-responsive micelles [65]. At physiological pH (7.4), the maleic anhydride groups were ring-opened by free amine groups from a protein (myoglobin) to form amide bonds. Ring-opening of the maleic anhydride moieties also liberated a free carboxylic acid group that could aid in complexation and stabilisation of the encapsulated protein. In this state, the polymer was able to form stable micelles. However, at pH values below 6.5, protonation of the amide group was thought to drive the reformation of the maleic anhydride ring, cleaving the amide bond. This resulted in de-stabilisation of the micelle and release of the protein [65].

A dialkyl maleic anhydride linker was also used by Gao et al. to attach pH-sheddable PEG chains to poly(L-lysine) and poly (L-glutamic acid) polyelectrolyte complex nanoparticles [63]. The labile amide bond could be cleaved at pH 6.5 to strip the PEG layer from the nanoparticles, leading to charge reversal and improved cell internalisation. Cellular internalisation was investigated on A549 cells by flow cytometry, which revealed that the nanoparticles pre-treated at pH 6.5 had a mean fluorescence intensity two times higher than nanoparticles pre-treated at pH 7.4 after 24 h due to reversal of the surface charge. Intracellular nanoparticle uptake was found to increase at prolonged pre-treatment times, attributed to a higher extent of shedding of the PEG layer [63].

In an interesting approach, Chen et al. grafted the side chain amine groups of PEG-*b*-poly(L-lysine) with a series of different anhydrides (DMMA, succinic anhydride, *cis*-aconitic anhydride and *cis*-cyclohexene-1,2-dicarboxylic anhydride) to produce block copolymers with gradient pH-responsivity [62]. Due to their slightly different chemical environments, the resulting amide bonds exhibited different sensitivities to acid-promoted hydrolysis. The rate of hydrolysis was determined to be fastest for the copolymers functionalised with DMMA, followed by *cis*-actonitic anhydride, then *cis*-cyclohexene-1,2-dicarboxylic anhydride. In agreement with the findings of Zhang et al. [9], the copolymer conjugated to succinic anhydride demonstrated no observable acid sensitivity. Release of doxorubicin from corresponding polymeric micelles followed the same trend, suggesting that the drug release profiles can be modulated based on the lability of the amide bond [62].

Hydrazone linkages are another means of imparting pH-responsivity to a system, as they are considered to be relatively stable at pH 7.4 but can undergo hydrolysis under acidic conditions. By exploiting this pH-dependent cleavage, coupled with the incorporation of *N*,*N*-diisopropylaminoethyl groups to further impart pH-responsivity, Liu et al. prepared thermo- and pH-responsive poly(L-aspartic acid)-based nanoparticles where PEG and doxorubicin were attached through cleavable hydrazone bonds to form a graft copolymer [66]. The grafted doxorubicin acted as the hydrophobic moiety to facilitate self-assembly of the nanoparticles, and also enabled encapsulation of additional doxorubicin within the hydrophobic core of the micelles through π-π stacking interactions. At pH 7.4, the nanoparticles released only 25% of doxorubicin after 50 h, while 60% release was observed at pH 5.0, which was mainly attributed to hydrolysis of the hydrazone linkages at low pH, releasing the doxorubicin and PEG components. Similar approaches have been used in a number of recent studies for pH-sensitive conjugation of doxorubicin to PEG-*b*-poly(L-aspartic acid) and poly(L-glutamic acid) derivatives [67,68,69].

Other pH-labile linkages, such as imine bonds, have also been investigated. Zhou et al. reported the preparation of thermo- and pH-responsive poly(γ-4-(propargoxycarbonyl)benzyl-L-glutamate) co-grafted with oligoethylene glycol and aldehydes [71]. Cross-linking of the grafted aldehyde groups with 1,6-hexanediamine facilitated the formation of nanoparticles with pH-labile imine cross-links that could be reverted back to linear polymer chains below pH 6.15. The cross-linked polymer was water soluble at pH values above 6.15 and between 0–100 °C, but exhibited a reversible lower critical solution temperature (LCST) at pH values below 5.8 [71].

Acetal groups represent another means by which pH-responsivity can be conferred to polypeptides. Takemoto et al. prepared a PEG-*b*-poly(L-aspartic acid) derivative conjugated with the anticancer drug gemcitabine through an acid-labile acetal linker [70]. Through a transacetalisation reaction, the authors were able to conjugate the drug at an overall content of ~10%. The release of gemcitabine was found to be accelerated at pH 4.2 (33.3%) compared to pH 7.4 (5.3%) after 72 h due to cleavage of the acetal group [70]. In a similar fashion, acid-mediated hydrolysis of ketal cross-linking groups has also been employed to trigger drug release from pH-responsive micelles composed of PEG-*b*-poly(L-aspartic acid)-*b*-poly(L-phenylalanine) [72].

### 2.3. pH-Responsivity Triggered by Changes to Polypeptide Secondary Structure

pH-responsivity can also be achieved by triggering changes in the secondary structure of polypeptides. Due to secondary interactions between the amino acid side chains and the peptide backbone, polypeptides can form complex secondary structures, such as α-helices, coils, and β-sheets [73]. These secondary structures are often determined by pH-dependent ionic or hydrophobic interactions, the disruption of which can drive changes in copolymer rigidity, membrane permeability or aggregation. For example, it is well established that poly(glutamic acid) exhibits a sharp helix-to-random coil transition with decreasing pH [74], while poly(lysine) undergoes random coil-to-helix transitions with increasing pH [75].

**Figure 5:**
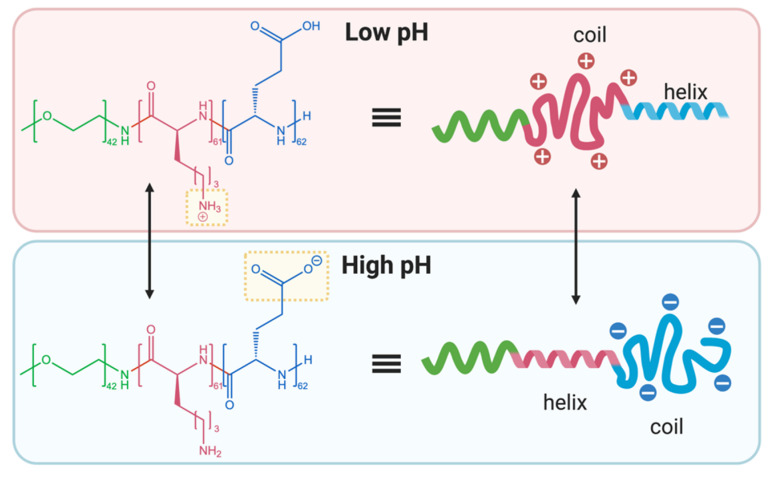
Structure of PEG-*b*-poly(L-lysine)-*b*-poly(L-glutamic acid) copolymer prepared by Schlaad and co-workers, showing corresponding changes to secondary conformation at low (1.9–3.1) and high (12.3–13.0) pH values (adapted from [76]).

Schlaad and co-workers exploited the opposing conformational transformations of poly(glutamic acid) and poly(lysine) with a pH-responsive PEG-*b*-poly(L-lysine)-*b*-poly(L-glutamate) copolymer [76]. At low pH (1.9–3.1), the poly(L-lysine) block existed predominantly in a random charged coil conformation, while the poly(L-glutamic acid) block adopted an α-helical conformation. At high pH (12.3–13.0), the opposite conformations were observed (i.e., α-helical poly(L-lysine) and randomly coiled poly(L-glutamate)) (Figure 5). β-sheet conformations were also observed at intermediate pH values (4.1 and 11.7). Under acidic conditions, the polymer preferentially aggregated into vesicles, but remained disassembled under basic conditions, depending on the location of the hydrophobic α-helix portion. Specifically, vesicle formation was preferred when the α-helix was flanked between the two hydrophilic blocks. At intermediate pH values, precipitation was observed as a result of complexation of the charged polypeptide components [76].

In 2017, Luan’s group prepared pH-sensitive PEG-poly(*ε*-caprolactone)-poly(L-glutamic acid) polymersomes loaded with doxorubicin [77]. This system demonstrated faster drug release at low pH values (5.0 and 6.5) compared to pH 7.4, which was reportedly the result of a charged coil-to-helix transition of the poly(L-glutamic acid) subunit, leading to increased copolymer rigidity, decreased membrane density and subsequently faster drug release. This charged coil-to-helix transition was also reported to be one of the primary mechanisms of pH-responsivity in PEG-*b*-poly(L-leucine)-*b*-poly(L-glutamic acid) pepsomes [78]. Similarly, Mostoufi et al. reported that the α-helical transition of PEG-*b*-poly(L-glutamic acid)-*b*-poly(L-leucine) and the subsequent shrinkage/relocation of the poly(L-glutamic acid) segment within the micelle was responsible for drug release under acidic conditions [79].

Tinajero-Díaz et al. synthesised two pH-responsive copolymers consisting of poly(ω-pentadecalactone)-*b*-poly(L-glutamic acid) (PDDL-poly(L-glutamic acid)) and poly(ω-pentadecalactone)-*b*-poly(L-lysine) (PDDL-poly(L-lysine)), respectively [80]. Circular dichroism revealed that both polymers formed α-helical conformations in their neutral, unionised states, but adopted randomly coiled conformations when ionised. For doxorubicin-loaded PDDL-poly(L-glutamic acid) nanoparticles, the ionic interaction between the doxorubicin and carboxylate side chain of the glutamic acid units were thought to be the primary mechanism of encapsulation. At pH 7.4, doxorubicin release plateaued at ~60% within the first 20 h, and no further release was observed even after 50 h. Contrastingly, drug release was accelerated at pH 2.0, releasing 100% of the drug within 5 h. This was potentially attributed to a helix-to-random coil transition mediated by pH-induced ionisation of the poly(L-glutamic acid) carboxylic acid groups, in combination with dissociation of the poly(L-glutamic acid)-doxorubicin ionic complexes [80].

Aside from pH-responsive nanoparticles, Zhao et al. described the development of a pH- and thermo-responsive hydrogel composed of methoxy PEG-*b*-poly(L-lysine)-*b*-poly(L-valine), which was found to have the optimal hydrophilic-lipophilic balance to facilitate the transition from a liquid to a gel (otherwise known as a sol-gel transition) [36]. On increasing the pH from 4.0 to 8.0, the gelation temperature was found to decrease from 38 °C to 27 °C due to deprotonation of the poly(L-lysine) amine groups and decreasing aqueous solubility of the polymer, promoting gel formation. The mechanism of this sol-gel transition was investigated further by Fourier transform infrared (FTIR) spectroscopy, which suggested that the proportion of β-sheet conformations within the polymer increased with increasing pH. At pH 7.4, dehydration of the PEG segment and changes to the secondary β-sheet conformation led to a synergistic effect in lowering the sol-gel transition temperature [36].

Chen et al. recently described an alternative approach towards the control of polypeptide secondary structures. pH-sensitive poly(L-lysine) vesicles with tuneable molecular assembly and membrane permeability were prepared by grafting the amine side chains of poly(L-lysine) with hexanoyl, decanoyl, or tetradecanoyl groups at 20 or 40% molar ratios [81]. In general, the peptide conformation could be tuned based on the length of the alkyl chain and the degree of substitution. For instance, poly(L-lysine) grafted with decanoyl (40 mol%) and tetradecanoyl (20 mol%) groups were found to change from α-helical and β-sheet conformations, respectively, to random coil conformations as the pH was decreased from pH 7.4 to 4.7. This change in conformation was coupled to an increase in the zeta-potential, caused by protonation of the poly(L-lysine) layers, which also acted to switch the membrane permeability of the vesicle. In agreement, the rate of doxorubicin release from the vesicles was found to accelerate with decreasing pH, partially due to increases in vesicle permeability resulting from conformational changes to the polymer [81].

### 2.4. pH-Responsivity Triggered by Supramolecular Interactions

Supramolecular pH-responsive polypeptide-based systems rely on non-covalent interactions to drive changes in polymer assembly and aggregation. This often involves electrostatic interactions between the charged side groups of a polypeptide with oppositely charged entities, such as ionised amino acids, drug molecules or inorganic ions. Typically, pH changes in the surrounding environment can lead to loss of these electrostatic interactions, leading to changes in the physicochemical characteristics of the system. However, other non-covalent interactions, such as hydrophobic interactions, can also be used to drive these changes. The appeal of supramolecular pH-responsive systems arises, in part, due to their relative ease of fabrication and dynamic nature.

**Figure 6:**
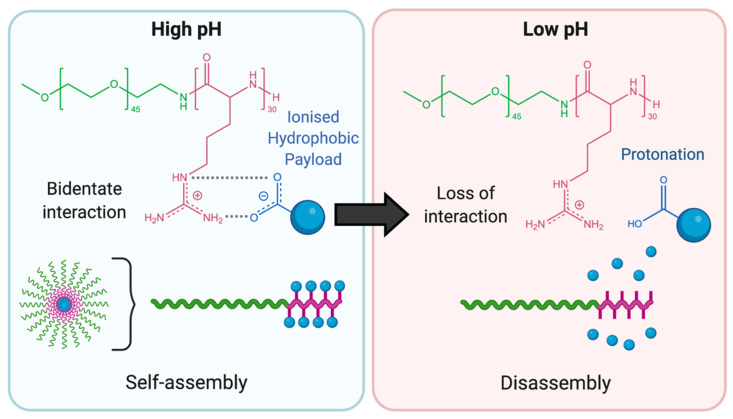
Schematic illustration of the bidentate interactions between the guanidinium moiety of PEG-*b*-poly(L-arginine) (pink) and the carboxylate group of the encapsulated hydrophobic payload (blue) reported by Praveen et al. (adapted from [82]).

For instance, Praveen et al. reported on the preparation of a PEG-*b*-poly(L-arginine) copolymer that could utilise the positively charged guanidinium groups of the peptide to form a bidentate interaction with the negatively charged carboxylate group of a dexamethasone derivative [82]. The PEG-*b*-poly(L-arginine) was able to self-assemble into nanoparticles through formation of a “supra-amphiphile,” where PEG formed the outer corona and the poly(L-arginine)-dexamethasone complex formed the inner core. Protonation of the carboxylic acid groups of the dexamethasone derivatives disrupted the stabilising bidentate interactions, resulting in disassembly of the aggregate. Nile Red dye was encapsulated in conjunction with the dexamethasone derivative to provide a visual marker of drug release. At pH 7.4, the supra-amphiphilic nanoparticles preserved 100% of the encapsulated Nile Red and dexamethasone, while complete released was observed at pH 5.0 (Figure 6).

Li et al. prepared supramolecular dendrimers based on electrostatic interactions between the positively charged amino groups of poly(L-lysine) and the negatively charged carboxylate moieties of glutamic acid-terminated poly(L-leucine) [83]. The result was the formation of a capsid-like nanoparticle, containing the glutamic acid-terminated poly(L-leucine) at the core, and the poly(L-lysine) dendrimer at the corona, attached to one another through non-covalent electrostatic interactions. Decreasing the pH to 6.2, to mimic the tumoral environment, was found to weaken these interactions due to protonation of the glutamic acid moieties, causing disassembly of the nanoparticle and release of the encapsulated drug [83]. Only 20% doxorubicin release was observed at pH 7.4 after 24 h, compared to approximately 70% at pH 6.2.

Similarly, using electrostatic interactions, Meng et al. prepared a series of pH-responsive supramolecular hydrogels composed of PEG-*b*-poly(L-glutamate) derivatives [84]. Addition of divalent Ca^2+^ caused a decrease in the temperature required for the sol-gel transition, resulting from coordination of the Ca^2+^ ions with the anionic carboxylate moieties of the poly(L-glutamate) block, effectively forming ionic cross-links. Accordingly, the hydrogels were found to demonstrate a lower sol-gel transition temperature at pH 7.0 compared to pH 4.0. Further, the formation of short fibrils at pH 7.0 was observed by atomic force microscopy (AFM) following the addition of Ca^2+^ at 50 °C, suggesting that hydrogel formation was driven by fibril self-assembly, which was in turn enhanced by the formation of ionic cross-links with Ca^2+^ and structural collapse on heating [84].

Rather than electrostatic interactions, Ni et al. employed hydrophobic interactions to drive the formation of supramolecular polypeptide-based nanodisks and nanosheets [85]. The authors prepared pH-responsive PEG-*b*-poly(L-glutamic acid)-*b*-poly(*N*-octylglycine) copolymers that could form disk-like structures at neutral pH, where the hydrophobic poly(*N*-octylglycine) block formed the core layer, while the poly(L-glutamic acid) and PEG blocks formed outer layers that flanked either side. Decreasing the pH to 4.8 significantly lengthened the structures, forming nano-sheets in the range of hundreds of nanometres. This finding was attributed to protonation of the poly(L-glutamic acid) block, which effectively increased its hydrophobicity and caused it to form part of the core layer [85].

## 3. Conclusions and Future Perspectives

pH-responsive polypeptides and polymer-peptide hybrids represent a promising class of materials for the development of novel drug delivery systems with tuneable physicochemical properties. These materials are ideal candidates because of their inherent biocompatibility, biodegradability, versatility, and their unique ability to form complex supramolecular structures. Numerous strategies have been explored to impart pH-responsivity to polypeptide-based systems, including the manipulation of ionisable functional groups, acid-labile linkages, changes to secondary structure, and supramolecular interactions. Their pH-responsive property allows these polymers to exploit physiological and pathological pH variations within the body to offer more efficient and targeted delivery of therapeutics. These technologies have potential markets across a wide range of biomedical applications, most notably in the development of biomaterials, sustained-release drug delivery systems, and tumour-targeting platforms.

While impressive advancements have been made in the preparation of pH-responsive polypeptide-based systems, the field remains in its infancy. Though a number of structurally diverse functionalities have been conjugated to polypeptides through post-polymerisation modifications (i.e., via click reactions with the polypeptide side chain), there are many pH-responsive functionalities with biologically relevant pK_a_ values that have not been investigated (e.g., quinoline, pyridine, and aniline derivatives). This represents an interesting area of research, as direct modification of synthetic polypeptides with such pH-responsive moieties has the potential to greatly simplify synthesis and design, and also offers exciting prospects in terms of tuneable pH-sensitivity. Additionally, although pH-responsivity is one means to improve drug delivery, future research will likely shift towards the preparation of dual- or multi-stimuli responsive systems, which can be designed to respond to multimodal triggers incorporating heat, redox, light, or enzyme responsivity. This will become particularly important for tumour-targeting applications, due to the relatively higher temperature of the tumour microenvironment, as well as the intracellular redox imbalance that exists within most cancer cells [86,87]. By developing systems that respond to multiple stimuli, it may be possible to enhance the efficacy of treatment and reduce off-target side effects, improving the overall quality of life of cancer patients. In the context of polypeptide-based nanoparticles, the attachment of active-targeting ligands may also lead to exciting improvements in cellular internalisation and treatment efficacy.

Additionally, it will become increasingly imperative to develop methods that offer precise control over amino acid sequence in high molecular weight polypeptides, which can allow for delicate manipulation of the supramolecular characteristics and overall morphology of assembled polymer systems. With a sound understanding of this relationship, polypeptide-based systems can be purposefully designed to form specific morphologies that enable biomimicry of complex proteins or viruses, broadening possibilities for drug and gene delivery. Finally, though many stimuli-responsive polypeptide-based systems exhibit promising physicochemical properties, their relatively complex synthesis and the absence of standardised manufacturing processes may make it difficult to attain regulatory approval, hindering their progression into the clinic. As many of these platforms remain in their pre-clinical stages, there is still much work to be done to better understand how these pH-responsive strategies fare when preparing materials for use in a clinical setting. Therefore, it is crucial that additional research is performed in this area to facilitate the successful clinical translation of these platforms.

## Figures and Tables

**Table 1:** Summary of key pH-labile linkages for the development of cleavable functionalities.

Representative Structure ^1^	pH-Responsive Component	Ref(s)
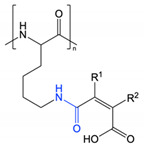	Amide	[9,61,62,63,64]
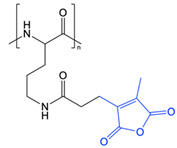	Anhydride	[65]
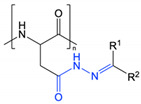	Hydrazone	[66,67,68,69]
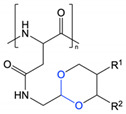	Acetal	[70]
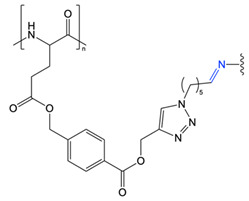	Imine	[71]
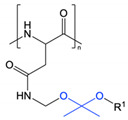	Ketal	[72]

^1^ pH-cleavable linker highlighted in blue.

## Data Availability

No new data were created or analysed in this study. Data sharing is not applicable to this article.
